# Microparticles of Lamivudine—Poly-ε-Caprolactone Conjugate for Drug Delivery via Internalization by Macrophages

**DOI:** 10.3390/molecules24040723

**Published:** 2019-02-17

**Authors:** Tomasz Urbaniak, Daniela Machová, Olga Janoušková, Witold Musiał

**Affiliations:** 1 Department of Physical Chemistry, Pharmaceutical Faculty, Wroclaw Medical University, Borowska 211, Wroclaw 50-556, Poland; tomasz.urbaniak@umed.wroc.pl; 2 Institute of Macromolecular Chemistry, Academy of Sciences of the Czech Republic v.v.i., Heyrovsky Sq. 2, 162 06 Prague 6, Czech Republic; danielamachova@imc.cas.cz (D.M.); janouskova@imc.cas.cz (O.J.)

**Keywords:** targeted drug delivery, drug-polymer conjugates, microparticles, drug delivery via phagocytosis, lamivudine, poly-ε-caprolactone

## Abstract

The past decade may be considered as revolutionary in the research field focused on the physiological function of macrophages. Unknown subtypes of these cells involved in pathological mechanisms were described recently, and they are considered as potential drug delivery targets. The innate ability to internalize foreign bodies exhibited by macrophages can be employed as a therapeutic strategy. The efficiency of this uptake depends on the size, shape and surface physiochemical properties of the phagocyted objects. Here, we propose a method of preparation and preliminary evaluation of drug-polymer conjugate-based microspheres for macrophage targeted drug delivery. The aim of the study was to identify crucial uptake-enhancing parameters for solid, surface modified particles. A model drug molecule—lamivudine—was conjugated with poly-ε-caprolactone via ring opening polymerization. The conjugate was utilized in a solvent evaporation method technique to form solid particles. Interactions between particles and a model rat alveolar cell line were evaluated by flow cytometry. The polymerization product was characterized by a molecular weight of 3.8 kDa. The surface of the obtained solid drug-loaded cores of a hydrodynamic diameter equal to 2.4 µm was modified with biocompatible polyelectrolytes via a layer-by-layer assembly method. Differences in the internalization efficiency of four particle batches by the model RAW 264.7 cell line suggest that particle diameter and surface hydrophobicity are the most influential parameters in terms of phagocytic uptake.

## 1. Introduction

**Scheme 1:**
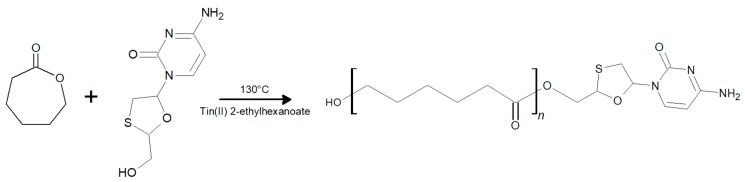
Ring-opening polymerization of ε-caprolactone initiated by lamivudine.

During the last decades the field of micro and nano drug delivery systems has gained unprecedented attention due to possibility of therapy improvement on various levels. Numerous advancements, e.g., side effect abatement, improvement of therapy efficiency, theranostic systems and control of drug distribution after administration are examples of the goals achieved through the use of particle-based submicron systems [1]. Delivery based on interactions between particles and cell-specific ligands is referred as active targeting, while taking advantage of leaky intercellular junctions, i.e., in inflamed or cancerous tissues, is termed passive targeting [2]. Crucial particle features such as size, chemical composition, targeting ligand type are selected depending on the addressed cells, and multiple chemical, physical or biological factors are considered during targeted drug delivery system design [3]. Among the numerous issues to be overcome in order to obtain efficient particle drug delivery systems, unwanted uptake by the phagocytic cell population seems to be one of the most serious [4]. Nevertheless, in the case of macrophage-related disorders, this phenomenon can be exploited as a drug delivery strategy. Such an approach was employed by research groups preparing drug delivery systems targeting macrophage populations infected by intracellular microbes such as *Leishmania* and *Toxoplasma* [5]. In addition to the hitherto confirmed contribution of macrophages to unspecific immunological responses and regulatory functions during inflammation [6], previously unknown subtypes of these cells, playing important roles in other pathological mechanisms, were described recently. A vital contribution of monocyte-derived cells was confirmed in tissue regeneration and scarification processes [7,8], fat metabolism [9], electric impulse conduction in the heart [10] and human immunodeficiency virus (HIV) resistance against multidrug therapy [11]. Monocyte-derived cells are found throughout the whole body, and like the other immune system cells, may be infected by HIV. The half-life of such infected cells is dramatically longer in comparison to other infected immune system cells such as CD4+ T helper cells. Therefore, macrophages are often recognized as viral reservoirs, especially these located in sites hardly accessible to drug substances, so called “sanctuaries”, i.e., in the central nervous system [12]. To address the HIV infected macrophage population, active antiretroviral drug delivery systems employing mannose as ligand were proposed [13,14]. The passive macrophage targeted drug delivery may exploit the ability of the cells to phagocyte foreign bodies, including microparticles [15]. The efficiency of phagocytic uptake is determined by the physiochemical properties of particle surface, and more specific interactions between molecules present on the particle surface and cell membrane receptors [4,16]. It should be taken into account that the macromolecules present in environment surrounding a particle may interact with the particle surface and modify its properties [17]. One of the approaches employed to control the fate of drug molecules after administration, is the widely employed strategy of covalent conjugation of the drug molecule with amino acid-derived ligands, antigens and synthetic molecules exhibiting specific affinity to target cells [18]. Other, nonspecific features provided by drug-polymer conjugation are passive targeting or protection from enzymatic activity [19]. In the case of macrophage targeted drug delivery, release from micro-matrices should be possibly postponed up to the onset of cellular uptake, for instance via conjugating a drug molecule with poorly hydrolyzable polyester chains like poly-ε-caprolactone (PCL). PCL is a biodegradable, non-toxic polyester synthesized mainly by ring-opening polymerization (ROP) of ε-caprolactone (CL). It’s characterized by slow degradation by polyester chain scission via a hydrolytic mechanism [20]. Weight loss during hydrolytic degradation of the polymer backbone varies according to the molecular weight, however it is often measured in days or even months. Products of the process contribute to the citric acid cycle, and are removed from the human body without negative effects [21]. Numerous investigated variants of ROP systems include metallic catalysts and initiators containing hydroxyl or amine moieties [22]. According to the suggested reaction mechanism, molecules acting as reaction initiators are built into the polymeric backbone via covalent ester bonds [23]. Application of drug tagged polymer as a component in microparticle preparation via the emulsion solvent evaporation method would result in delayed drug release. In order to achieve high phagocytic uptake, the formed microspheres should be characterized by beneficial values of particle zeta potential, hydrophobicity, size, and possibly may contain molecules specifically interacting with target cell receptors [4,24,25]. The physiochemical properties of the sphere surface determine the uptake efficiency, and may be modified via layer-by-layer (LbL) deposition techniques [26]. LbL polyelectrolyte sorption enables modification of particle surface charge as well as the introduction of surface chemical groups for further modification, for example click-chemistry ligand bioconjugation. Polyelectrolyte shells can be composed of any macromolecule charged at a given pH [27], such as dextran (DEX), heparin (HEP) or poly(allylamine hydrochloride) (PAH). Unmodified PCL cores are characterized by high hydrophobicity, often beneficial in terms of phagocytosis [28]. In contrast polyelectrolyte coatings may provide non-zero zeta potential values, which are favored by phagocytes; moreover, the polyelectrolyte may determine the type of macromolecule(s) adsorbed on a particle surface after exposure to a biological medium [29]. Specific affinity of various biopolymers to phagocytic cell surface receptors was suggested hitherto, and might influence overall uptake efficiency [30]. The aim of presented study was to conjugate a model antiretroviral drug–lamivudine (LV)—with a low molecular weight PCL chain in a ROP reaction catalyzed by tin(II) 2-ethylhexanoate (SO, Scheme 1).

**Figure 1:**
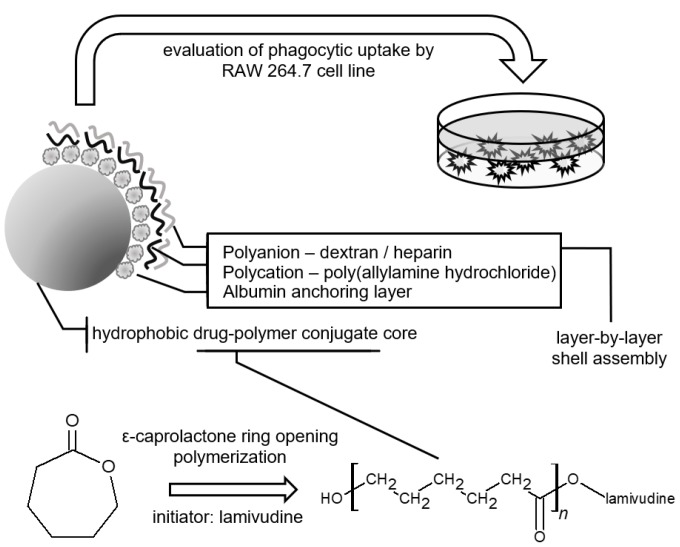
Schematic conception of the development of the lamivudine-poly-ε-caprolactone conjugate particles for intra-macrophagic drug delivery.

Subsequently, the synthesis product was utilized in the preparation of drug-polymer conjugate (PCL-LV) based micro-matrices with surfaces modified via a LbL polyelectrolyte deposition technique. LV is one of the first-regimen antiretroviral drugs prescribed in the case of HIV infection. The evaluated drug delivery system can be potentially utilized as a passive targeting system aiming HIV-infected macrophages via local micro-suspension administration, or can serve as a basis for more selective active targeting strategies after ligand surface bioconjugation. Herein, we present the results of preliminary particle-cell interaction experiments revealing the influence of the particle surface physiochemistry on uptake efficiency (Figure 1).

## 2. Results

### 2.1. Conjugate Synthesis

**Figure 2:**
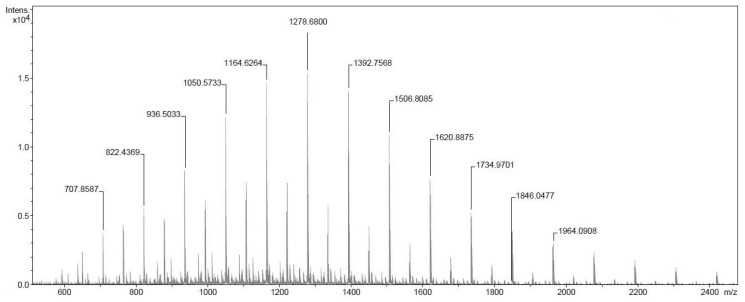
Mass spectra of synthesized lamivudine–poly-ε-caprolactone conjugate.

**Figure 3:**
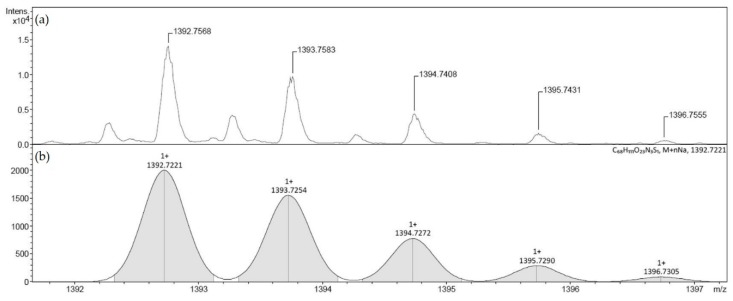
Isotopic distribution for positively charged conjugate coupled with sodium ion (**a**) and spectra simulation of conjugate composed of ten subunits, and drug molecule with singular sodium adduct (**b**).

The mass spectra of the ROP product (Figure 2) contain families of peaks, where each family corresponds to a particular type of polymeric molecule differing in molecular mass. The specific family of caprolactone derivatives is indicated by a repeating *m*/*z* interval equal to the molecular weight of a single poly-ε-caprolactone-mer. Detailed views of the isotopic distribution of the high intensity family of peaks with corresponding software simulation for the drug-polymer conjugate are presented on Figure 3a,b.

**Figure 4:**
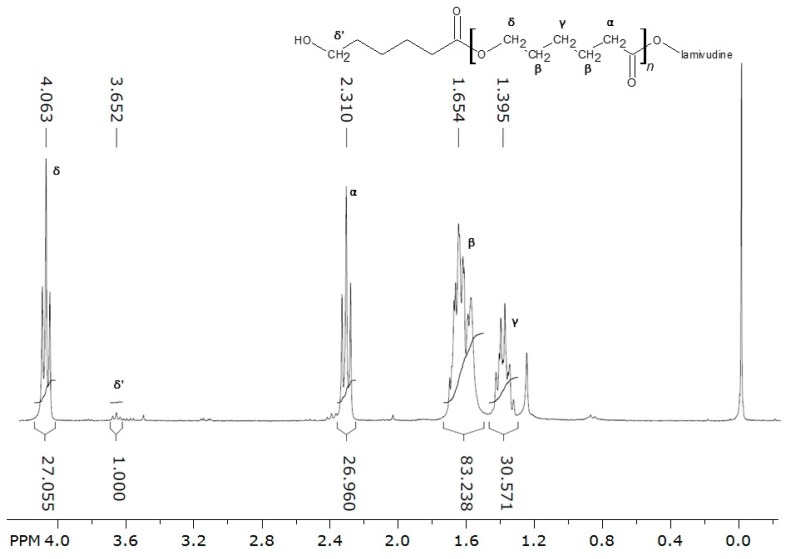
^1^H-NMR spectra of lamivudine-poly-ε-caprolactone conjugate with protons assigned to respective peaks.

Peaks observed on the ^1^H-NMR spectrum (300 MHz, CDCl_3_) of the synthesized conjugate are as follows: δ = 4.06 (t, CH_2_), 3.65 (t, CH_2_), 2.31 (t, CH_2_), 1.65 (m, CH_2_), 1.39 (m, CH_2_). Signals were assigned to individual protons (Figure 4). An average molecular weight of 3313 Da was calculated from the ratio of integrations of end group hydrogens (Figure 4, δ’) to polymeric backbone hydrogens (Figure 4, δ).

**Figure 5:**
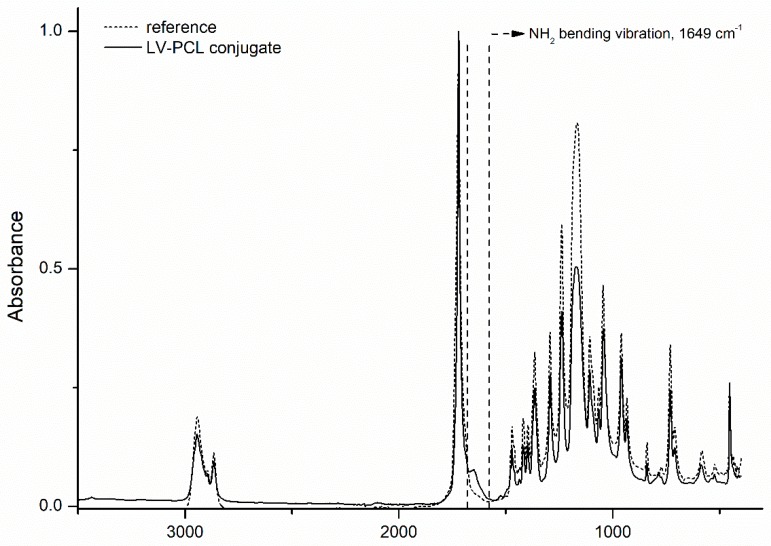
IR spectra of and polymer-drug conjugate and non-conjugated poly-ε-caprolactone reference.

Conjugate and reference polymer IR spectra in the 3500–400 cm^−1^ region (Figure 5) display the following bands: 2949 cm^−1^ asymmetric CH_2_ stretching, 2865 cm^−1^ symmetric CH_2_ stretching, 1727 cm^−1^ carbonyl stretching, 1240 cm^−1^ asymmetric COC stretching, 1190 cm^−1^ OC-O stretching, 1170 cm^−1^ symmetric COC stretching. In the conjugate spectra a distinctive symmetric NH_2_ bending vibration was observed at 1649 cm^−1^.

### 2.2. LbL Shell Assembly and Particle Characterization

**Figure 6:**
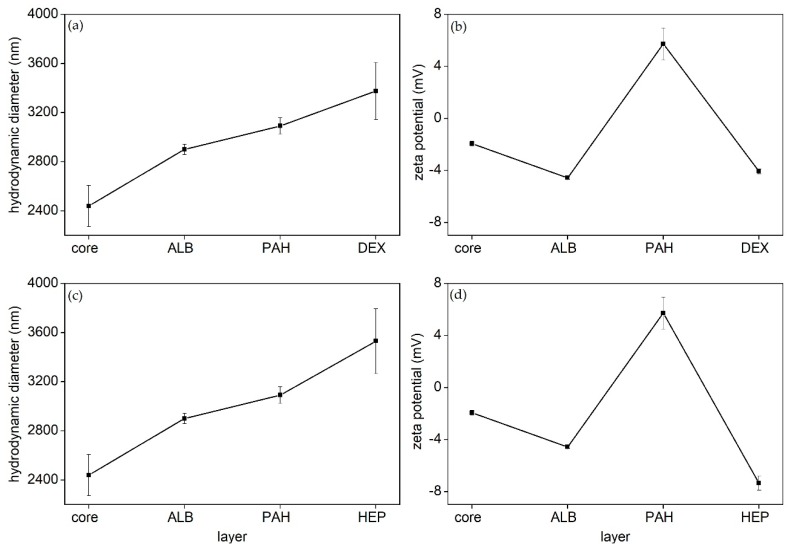
Hydrodynamic diameter increase (**a**), (**c**) and zeta potential changes (**b**), (**d**) during deposition of subsequent polyelectrolyte layers.

Subsequent deposition of polyelectrolyte layers on conjugate solid cores was confirmed by monitoring the variability of surface zeta potential and increase of particle hydrodynamic diameter (Figure 6). LV-PCL cores had a hydrodynamic diameter of approximately 2.4 µm and slightly negative zeta potential derived from drug molecule end groups and ester group carbonyl oxygens bearing slightly negative charges. Lipophilic cores covered with BSA exhibited negative surface charge; subsequent deposition of a PAH layer resulted in a zeta potential increase to positive values, whereas deposition of the polyanions HEP and DEX endowed the particles with negative surface charges. The hydrodynamic diameter of the particles increased continuously during the deposition of subsequent layers.

### 2.3. Scanning Electron Microscopy Imaging

**Figure 7:**
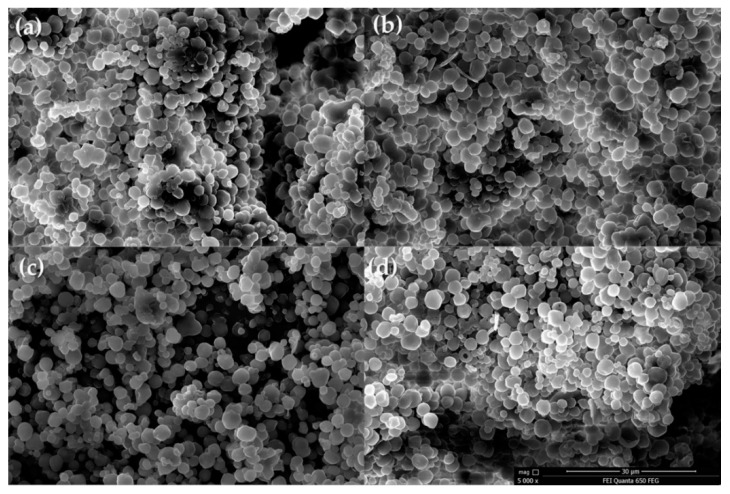
Scanning electron micrographs of obtained structures: particles with poly(allylamine hydrochloride) (**a**), heparin (**b**) and dextran (**c**) outer layers and pure cores (**d**).

The morphology of the prepared structures was evaluated via SEM imaging (Figure 7). The obtained particles are spherical with a moderately smooth surface. No significant difference in size of particle cores and dry coated particles was observed.

### 2.4. Phagocytosis In Vitro Assay

**Figure 8:**
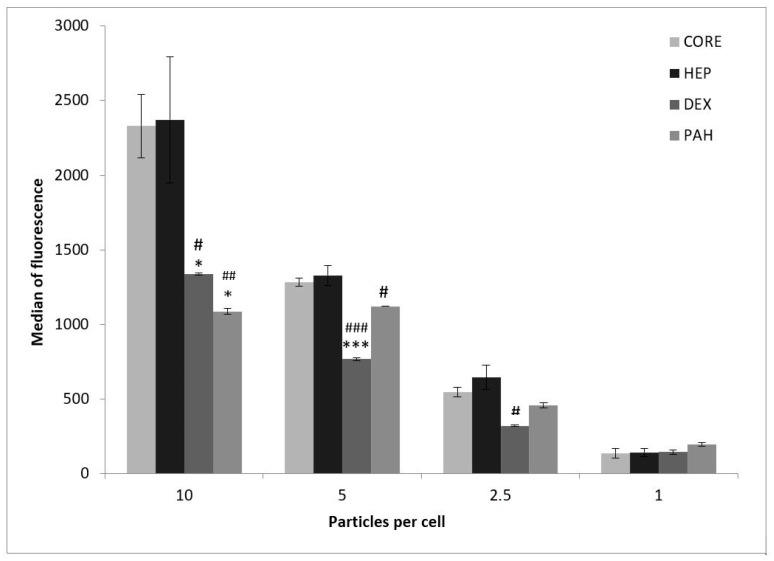
Intracellular presence of particles with outer-layers composed of dextran (DEX), heparin (HEP), poly(allylamine hydrochloride) (PAH) and nonmodified conjugate cores (CORE) labeled by fluorescent coumarin-6 in RAW 264.7 cells. Cells were incubated with the particles for 2 h before flow cytometry assay. The one-way ANOVA test followed by Tukey’s test was used to compare the effectiveness of internalization of nonmodified conjugate core with particles in the same concentration (*** *p* < 0.001, ** *p* < 0.01, and * *p* < 0.05) and compare the effectiveness of internalization heparin coated particle with other particles in the same concentration (### *p* < 0.001, ## *p* < 0.01, and # *p* < 0.05).

Fluorescence derived from internalized particles was observed in experiments employing four different particle to cell ratios. Median of fluorescent intensity (MFI) was not significant different at a 1:1 ratio for all particles and in case of DEX at a 2.5:1 ratio compared with untreated cells. The intensity of the fluorescence increased with the increase of particle to cell ratio. Cells incubated at 10:1 and 5:1 ratios exhibited significant differences in fluorescence intensity compared with untreated cells, depending on the particle type (Figure 8). The highest values of MFI were attained in the case of pure solid cores and particles coated by HEP in a 10:1 ratio. Pure solid core and HEP-coated particles showed significant higher uptake than particles coated with DEX and PAH. Pore solid cores showed significant higher uptake than particles coated with DEX (* *p* < 0.05) as well as in the case of particles coated with PAH (* *p* < 0.05). HEP-coated particles showed the highest uptake properties in comparison with particles coated with DEX (# *p* < 0.05) and particles coated with PAH (## *p* < 0.05).

### 2.5. In Vitro Viability Assay

**Figure 9:**
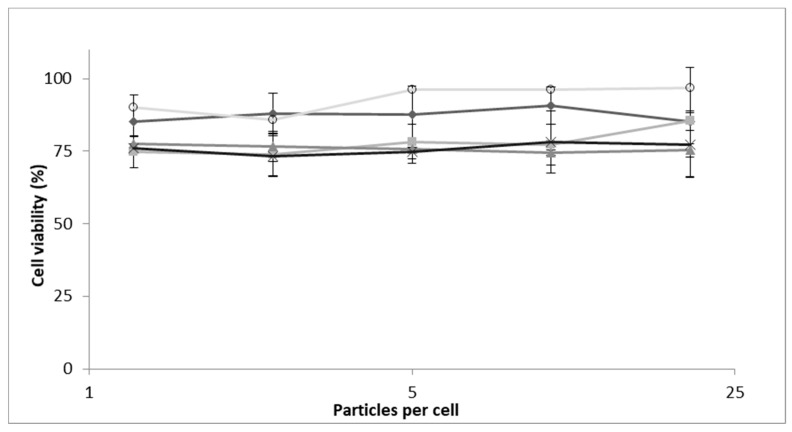
Cytotoxicity of particles with outer-layers composed of dextran (♦), heparin (■), poly (allylamine hydrochloride) (▲) and nonmodified conjugate cores (×) determined using the Alamar Blue Assay. Untreated cells were used as a control (○). The results are shown as the mean cell viability ± SD.

None of the particles caused toxicity effects in Raw 264.7 cells (Figure 9). The viability of cells was not significantly influenced in comparison with cells which were not incubated with particles.

## 3. Discussion

### 3.1. Conjugate Synthesis

Formation of LV-PCL conjugate was confirmed via spectroscopic methods. The mass spectrum of the obtained product is characteristic for polymeric molecules. It exhibits peak sets separated from each other by a *m*/*z* value of 114 Da, equal to the molecular weight of a PCL monomer unit. The isotopic distribution of peaks, in the set with highest intensities, matches the distribution simulated by software for a positively charged LV-PCL conjugated with sodium adduct (Figure 2). Thus, we confirmed the presence of LV-PCL conjugate in the analyzed samples. Infrared spectra of pure polymer and the obtained conjugate overlap and the observed vibrations are consistent with the pure polymer spectra described by Elzein et al. [31]. A distinctive symmetric NH_2_ bending vibration at 1649 cm^−1^ in the conjugate spectra suggests the presence of lamivudine-derived amine groups. The ^1^H-NMR spectra confirm the polymeric structure of the obtained product. The molecular weight value calculated from the ^1^H-NMR spectra of 3.13 kDa, close to the 3.82 kDa molecular weight obtained by gel permeation chromatography (GPC) analysis. The consistence of these values with the Mw of 3.76 kDa, predicted from the polymerization reactants ratio, confirms the occurrence of ROP initiated by the drug molecule.

### 3.2. Microparticle Formation and LbL Shell Assembly

The product obtained by a solvent evaporation method was evaluated in DLS experiments. The obtained solid particle cores with hydrodynamic diameter of 2.44 µm exhibited a surface zeta potential of −1.94 mV. Further modification of the particle surface with oppositely charged polyelectrolytes led to changes in the particle surface charge, consistent with the charge exhibited by polymers at pH 7.4, the albumin (pI = 4.9), heparin and dextran were negatively charged, whereas poly(allylamine) hydrochloride was positively charged under the applied conditions (Figure 6). Subsequent deposition of polymer layers was simultaneously indicated by an increase of hydrodynamic diameter up to 3.53 µm for particles with heparin outer layers and up to 3.34 µm for dextran outer layers. SEM imaging confirmed the formation of spherical structures. Notably, the particle diameters were lower than the hydrodynamic diameters obtained in DLS experiments. The increase in hydrodynamic diameter after deposition of subsequent layers was not visible in SEM measurements. In a solvated state particles covered with biopolymers exhibited differences in hydrodynamic diameter, which strongly depends on hydration of the surface polyelectrolytes in the swollen state. These differences occurred due to the fact that particles were in a dry state under vacuum during SEM imaging, as well as to the different principles on which the employed techniques are based [32].

### 3.3. In Vitro Phagocytosis assay

Various factors influence the rate of particle phagocytosis by macrophages. Particle size, shape and surface charge play significant roles [29,33]. The particle-environment and particle–cell interaction determined by specific properties of a particle outer layer may lead to promotion or inhibition of particle internalization [34]. Bead-shaped structures are usually produced, and often assessed as drug delivery micro-structures. Spherical particles are considered as favored by macrophages, according to the high compatibility of the spheres with cell membrane surface curvature during phagocytosis [35]. The particles applied to the cells at 1:1 ratio didn’t influence significantly the variability of fluorescence intensity of particles in evaluated cells (Figure 8). At higher particle:cell ratios differences between the phagocytosis rates of particles with different surface compositions were observed. The highest 10:1 ratio of particles to cells resulted in a significantly higher phagocytosis rate for pure conjugate cores and particles with a heparin outer layer. The same outcome was observed in case of lower ratios (1:5 and 1:2.5), but was less pronounced. Conjugate cores and HEP-coated particles entered the cells most efficiently, and exhibited similar cytotoxicity in the cell viability assay as PAH and DEX-coated particles, which were internalized less efficiently (Figure 9). We aimed to verify that the enhanced cellular uptake of non-modified cores and particles with HEP outer layers is not caused by its cytotoxicity and the subsequently higher permeability of dying cells. Presented data suggest that all types of particles, which are not cytotoxic, are suitable carriers for antiviral drugs. The non-coated particles exhibited low surface zeta potential, however they were phagocyted efficiently, possibly due to their favorable hydrodynamic diameter of ca. 2.4 µm, and high hydrophobicity. The diameters of described particles differ by up to 1 µm depending on the number of shell layers. According to previously reported data, model particles of diameter ca. 2 µm are phagocyted most efficiently [16,29,35,36]. The highly hydrophobic surface of the cores could also contribute to the observed results due to the strong interaction between the macrophage surface and the hydrophobic surfaces of particles [28]. Particles with outer layers of DEX or PAH exhibited the poorest internalization. The presence of fetal bovine serum in the experiment environments, which partly reflects an in vivo environment, introduces dozens of proteins which interact with particle surfaces and contribute to further particle-cell interactions [25]. Due to the high concentration of negatively charged albumin in the cell growth media, adsorption on the positively charged PAH-coated particle surface is a possible scenario [37]. Albumin -oated particles are known as poorly-phagocyted variants of modified particles, due to the hydrophilic nature of their surfaces [29]. This observation correlates with the absence of phagocytosis rate increase between the two highest particle:cell ratios observed in our experiments. Reported applications of dextran as a surface modifier resulted in elevated macrophage uptake, what was ascribed to the activity of scavenger receptors and to mannan-binding lectins [38]. Our data indicate a lower phagocytosis rate in comparison to hydrophobic cores and heparin-coated particles, however a phagocytosis efficiency increase was observed with increased particle:cell ratio, unlike in the case of PAH-coated particles. Heparin employed as particle outer layer and as coating, is widely used as a cell response alleviating agent [39]. In contrast, our data indicate a high particle uptake after heparin modification. Notwithstanding, there are reports of opposite cell reactions, linked with macromolecules present in the cell growth medium during experiments. It was shown that the presence of heparin in the cell environment triggers phagocytic uptake by mice macrophages [17]. Moreover, it was concluded that adsorption of the heparin on the particle surface is crucial in this phenomenon. We assume that the increased uptake may be correlated to heparin scavenger receptor affinity [40,41]. Therefore, it can be proposed that specific interactions between particle surfaces and receptors resulted in the observed high uptake of heparin-coated particles. This effect may be cell-line dependent, due to the interspecific variability of surface receptor expression.

## 4. Materials and Methods 

### 4.1. Materials

ε-Caprolactone (Sigma Aldrich, Darmstadt, Germany) was distilled under reduced pressure prior to use, stored under reduced pressure. Other reagents were sourced as follows: lamivudine (Sigma Aldrich), tin(II) 2-ethylhexanoate (Sigma Aldrich), Mowiol (Roth, Zielona Góra, Poland), dichloromethane (Chempur, Piekary Śląskie, Poland), methanol (Chempur), CDCl_3_ (Sigma Aldrich), THF for HPLC (Chempur), polystyrene standards (Sigma Aldrich), acetonitrile MS (Sigma Aldrich), dextran (Sigma Aldrich), bovine serum albumin (Sigma Aldrich), poly(allylamine hydrochloride) (Sigma Aldrich), heparin sodium salt from porcine intestinal mucosa (Sigma Aldrich), 6-coumarin (Sigma Aldrich), Alamar Blue^®^ cell viability reagent (Life Technologies, Prague, Czech Republic), Dulbecco’s modified Eagle’s medium (DMEM) supplemented with 100 units of penicillin and 100 µg · ml^−1^ streptomycin (Life Technologies), RAW 264.7 cell line (LGC standards - ATCC, Kiełpin, Poland).

### 4.2. Conjugate Synthesis

The ring opening polymerization procedure was performed in three necked flask equipped with a magnetic stirrer, at 130 °C under a dry nitrogen atmosphere for 4 h. The molar ratio of reactants was as follows: EC:LV:SO 31:1:8763. Crude product was dissolved in dichloromethane and recrystallized from cold methanol, dried, characterized and stored under vacuum until further use.

### 4.3. Microsphere Preparation

Conjugate solid particle cores were obtained via an o/w emulsion solvent evaporation method. Emulsions were obtained by homogenization of 100 mg/mL conjugate and 1 µg/mL the fluorescent dye coumarin-6 dissolved in dichloromethane (phase A) with 1 *w*/*w*% polyvinyl alcohol dissolved in distilled water (phase B). In order to obtain an emulsion, the two immiscible phases A and B at a ratio of 1:10 were homogenized for two minutes at 20,000 rpm with a PRO250 laboratory rotor-stator homogenizer (Bioeko, Siepraw, Poland). Subsequently, the emulsion was stirred for two hours in room temperature at a speed of 350 rpm in order to evaporate the organic phase. Resulting particle suspensions were centrifuged, rinsed with water, dried and stored in a desiccator until further use. 

### 4.4. Layer-by-Layer Shell Assembly

An initial albumin layer was deposited to enable further sorption of charged polyelectrolytes on the surface of hydrophobic LV-PCL cores. Lipophilic superficial domains of albumin (ALB) enable its attachment to uncharged surfaces, and provide an initial charge for further LbL assembly. The following coatings were prepared: ALB/PAH, ALB/PAH/HEP and ALB/PAH/DEX. Subsequent layers were deposited from phosphate buffer saline solutions at the following concentrations: 2 mg/mL of DEX, HEP and ALB, 0.5 mg/mL of PAH. Deposition occurred during 15 min of core exposure to polyelectrolyte solution, with PBS washing between subsequent sorption steps.

### 4.5. Product Characterization

The formation of the drug-polymer conjugates in ring opening polymerization was evaluated by electrospray mass spectrometry in acetonitrile on a micrOTOF-Q mass spectrometer (Bruker Daltonics, Bremen, Germany). The isotopic distribution of the peaks corresponding to functionalized polymeric chains found by the experiment was compared to the patterns simulated by the DataAnalysis 3.4 software (Bruker). FTIR spectra were performed on a Nicolet iS50 FTIR (Thermo Scientific, Waltham, MA, USA) spectrometer by using an attenuated total reflection (ATR) module. The spectra were scanned over a wavenumber range of 3500–400 cm^−1^. The proton nuclear magnetic resonance spectra were obtained using an ARX 300 MHz NMR spectrometer (Bruker, Billerica, MA, USA) in chloroform-d (CDCl_3_). Chemical shifts are given in ppm units. Signal multiplicities are represented by the following abbreviations: s (singlet), d (doublet), t (triplet), and m (multiplet). Molecular weight of sample was determined by room temperature GPC analysis performed in tetrahydrofuran on a model Ultimate 3000 HPLC system (Thermo Scientific) equipped with a Phenogel 103 Å column. Obtained values were based on polystyrene standard calibration, and corrected according to a Mark-Houwink factor equal 0.56 [42]. The morphology of microspheres was observed under scanning electron microscopy. Samples were covered by a fine gold layer and observed on a Quanta 650 microscope (15 kV, (FEI, Hillsboro, OR, USA). Hydrodynamic diameter and zeta potential were measured in phosphate buffer saline on Zetasizer Nano apparatus (Malvern, Worcestshire, UK) at 25.0 ± 0.1 °C. At least three measurements were carried out for each measurement.

### 4.6. Cell Culture

RAW 264.7 cell line (ATCC, Poland) was cultured in Dulbecco’s modified Eagle’s medium (DMEM) supplemented with 100 units of penicillin and 100 µg · ml^−1^ streptomycin (Thermo Fischer Scientific, Prague, Czech Republic) in 25 cm^3^ flasks and cultivated in a humidified incubator at 37 °C with 5% CO_2_. 

### 4.7. Quantification of Intracellular Trafficking of Fluorescently Labeled Particles via Flow Cytometry

Cells were incubated in 24-well plate with solid core particle for 2 h in 5% CO_2_ at 37 °C. The amount of fluorescently labeled particles was calculated as particles per cell. After washing with PBS, 2 × 10^5^ cells were gently mechanically harvested, centrifuged and resuspended in 0.5 mL of PBS with 0.5% BSA. FACS Verse (Becton Dickinson, Franklin Lakes, NJ, USA) was used to analyze the samples (10 × 10^3^ cells per sample), and data were processed using FlowJo software V7.6.1 (FlowJo, Ashland, OR, USA). The median of the fluorescence intensity (MFI) of exclusively live cell populations incubated with solid core particles with different polyelectrolyte shells was determined. Dead cells were marked by 7-Aminoactinomycin D (Abcam, Cambridge, UK). Cells that were not incubated with microspheres were used as a negative control. All samples were measured in duplicate in three independent experiments. 

### 4.8. Cell Viability Assay

Cytotoxicity was determined using Alamar Blue^®^ cell viability in RAW 264.7 cells according to the manufacturers protocol. Cells were seeded at density of 5000 cell/well in 100 µL of media into 96-well plate 24 h prior to treatment. The medium was then replaced by 100 µL of a serial dilution of particles in complete cell culture medium. The macrophages were subsequently incubated for 72 h in 5% CO_2_ at 37 °C. Then, 10 µL of Alamar Blue reagent was added to each well and incubated for 4 h in 5% CO_2_ at 37 °C. The active compound of Alamar Blue reagent, resazurin, was reduced to the highly fluorescent compound resorufin only in viable cells. The fluorescence intensity of resorufin was measured using a Synergy Neo plate reader (Bio-Tek, Prague, Czech Republic) at 570 nmEx/600 nmEm. Non-treated cells were used as a positive control. All samples were measured in triplicate in three independent experiments.

### 4.9. Statistical Analysis

In vitro results are presented as average ± standard deviation (SD). The one-way ANOVA test was used to determine the significant difference of the result. The ANOVA followed by Tukey’s test was used to compare difference between two groups and all statistical analysis was performed using Prism v5 software (GraphPad, San Diego, CA, USA). A value of *p* < 0.001, *p* < 0.01, and *p* < 0.05 was considered statistically significant for comparison of the effectiveness of internalization of nonmodified conjugate core with another particles in the same concentration (*** *p* < 0.001, ** *p* < 0.01, and * *p* < 0.05) and the effectiveness of internalization heparin coated particle with another particles in the same concentration (### *p* < 0.001, ## *p* < 0.01, and # *p* < 0.05).

## 5. Conclusions

A drug-polymer conjugate were synthesized via ROP of ε-caprolactone with lamivudine employed as initiator. The obtained product was utilized as substrate material for formation of solid 2.4 µm particles in a solvent evaporation method. Particle surface modification with biodegradable polyelectrolytes was achieved via a LbL deposition method. The degree of microsphere phagocytosis by a model macrophage RAW 264.7 cell line varied depending on the outer layer composition. The obtained results suggest complex particle-cell interactions influenced by particle size, hydrophobicity, specific receptor-based affinity and particle-environment interactions. As the polyelectrolyte surface modification did not improve uptake significantly, the particle hydrophobicity and diameter were identified as crucial for microparticle uptake efficiency.
